# Diversity and taxonomy of *Phragmidium* on *Rosa* in China, with descriptions of two new species

**DOI:** 10.3897/mycokeys.138.201984

**Published:** 2026-08-07

**Authors:** Yun Liu, Zelin Zhao, Zhimin Du, Xiuhan Xing, Ning Jiang, Yoshitaka Ono, Yingmei Liang

**Affiliations:** 1 College of Agricultural Science and Technology, Shandong Agriculture and Engineering University, Jinan 250100, China Faculty of Education, Ibaraki University Ibaraki Japan; 2 Key Laboratory of Forest Protection of National Forestry and Grassland Administration, Institute of Forest Ecology, Environment and Nature Conservation, Chinese Academy of Forestry, Beijing 100091, China College of Agricultural Science and Technology, Shandong Agriculture and Engineering University Jinan China; 3 Faculty of Education, Ibaraki University, Ibaraki 310-8512, Japan Key Laboratory of Forest Protection of National Forestry and Grassland Administration, Institute of Forest Ecology, Environment and Nature Conservation, Chinese Academy of Forestry Beijing China; 4 Institute of Life and Environmental Sciences, University of Tsukuba, Ibaraki 305-8572, Japan Museum of Beijing Forestry University, Beijing Forestry University Beijing China; 5 Museum of Beijing Forestry University, Beijing Forestry University, Beijing 100083, China Institute of Life and Environmental Sciences, University of Tsukuba Ibaraki Japan

**Keywords:** Multi-gene phylogeny, new taxa, *
Phragmidium
*, species diversity, taxonomy

## Abstract

The rust genus *Phragmidium* (*Phragmidiaceae*, *Pucciniales*) comprises significant plant pathogens infecting *Rosaceae*, particularly species of *Rosa*. Although numerous *Phragmidium* taxa have been recorded on *Rosa* in China, their evolutionary relationships and true species diversity remain poorly resolved. Here, a systematic summary of Chinese *Rosa*-associated *Phragmidium* is presented by integrating detailed morphological examinations with multilocus phylogenetic analyses of ITS and LSU sequence data, using extensive specimens deposited in major international fungaria and herbaria. Two novel species, *P. shennongjiaense* and *P. xinjiangense*, are described and illustrated, and the phylogenetic affinities of 21 previously accepted species are clarified based on updated taxonomic accounts. This work underscores the indispensability of an integrative taxonomic framework—coupling morphological nuances with multilocus datasets—for accurately delineating species boundaries and resolving the rich cryptic diversity of rust fungi in East Asia.

## Introduction

The rust genus *Phragmidium* Link (*Phragmidiaceae*, *Pucciniales*) comprises autoecious, macrocyclic or microcyclic fungi that are obligate biotrophic parasites mainly on members of *Rosaceae*, especially species of *Rosa*, *Rubus*, and *Potentilla* (Arthur 1934; Gäumann 1959; Hiratsuka et al. 1992; Wahyuno 2002; Cummins and Hiratsuka 2003; Zhuang et al. 2012; Liu 2021). Species of *Phragmidium* occur on both cultivated and wild rosaceous plants and may have notable economic and ecological effects. On cultivated roses, severe infections can lead to premature defoliation, reduced plant vigor, and loss of ornamental and commercial value. On wild hosts, rust infection may influence plant growth, reproductive performance, and population dynamics (Pscheidt and Rodriguez 2016).

*Phragmidium* was established by Link (1816) and has long been treated as a distinct rust genus associated with *Rosaceae*. Early taxonomic studies delimited species mainly on the basis of spore morphology, life cycle characters, and host association (Winter 1884). Müller (1886) provided one of the earliest detailed accounts of *Phragmidium* species on *Rosa* and *Rubus*, and the monograph by Sydow and Sydow (1915) later provided a more consolidated treatment of the genus. Subsequent works by Arthur (1934), Gäumann (1959), and Cummins and Hiratsuka (2003) further clarified the use of morphological characters in rust taxonomy. Even so, species recognition in *Phragmidium* remains problematic. Many species have overlapping teliospore dimensions, similar numbers of teliospore cells, and comparable patterns of spore wall ornamentation. Morphological variation related to host species, developmental stage, or environmental conditions may also blur species boundaries. More than 110 species of *Phragmidium* have been described worldwide, and East Asia has been regarded as one of the main centers of diversity of the genus (Wei 1988; Hiratsuka et al. 1992; Cao and Li 1999; Zhuang et al. 2012; Liu 2021; Sun et al. 2022; Zhang et al. 2025). China is especially rich in *Phragmidium* species. Earlier studies on Chinese rust fungi were based largely on morphology, host records, and herbarium specimens (Teng 1963; Tai 1979; Wei 1988; Cao and Li 1999; Zhuang et al. 2012). At present, approximately two dozen species have been reported from *Rosa* hosts in China, including *P. rosae-multiflorae* Dietel, *P. tuberculatum* Jul. Müll., *P. mucronatum* (Pers.) Schltdl., *P. fusiforme* J. Schröt., and *P. butleri* Syd. & P. Syd. (Wei 1988; Zhuang et al. 2012; Liu et al. 2020; Liu 2021; Sun et al. 2022; Zhang et al. 2025). However, the species limits and phylogenetic relationships of many Chinese taxa remain uncertain. This uncertainty is largely caused by limited sampling, incomplete observations of life cycle stages, reliance on host association in some earlier records, and the absence of molecular data for many described species.

Molecular evidence has become an essential component of rust fungal taxonomy. The internal transcribed spacer region (ITS) and the large subunit nuclear ribosomal RNA gene (LSU) are among the most frequently used markers because they are available for many fungal groups and allow comparison with existing sequence data (Aime 2006; Aime et al. 2006, 2018). ITS was proposed as the universal fungal DNA barcode because of its relatively high interspecific variation (Schoch et al. 2012), whereas LSU is more conserved and is informative for deeper phylogenetic relationships. In *Phragmidium*, molecular data are particularly valuable because closely related or morphologically similar species may occur on related *Rosa* hosts, and diagnostic morphological characters are often subtle. The combined analysis of ITS and LSU can therefore provide an independent test of morphology-based species delimitations. Recent studies and field surveys in China have continued to reveal previously unrecognized rust fungi (Zhao et al. 2021; Sun et al. 2022; Zhang et al. 2025), suggesting that the diversity of *Phragmidium* on *Rosa* remains incompletely documented.

In this study, *Phragmidium* species occurring on *Rosa* in China were reassessed using morphological characters and molecular phylogenetic evidence. Specimens deposited in major fungaria and herbaria were examined, and phylogenetic analyses were conducted using a combined ITS and LSU dataset. The objectives were to clarify the species boundaries of Chinese *Phragmidium* species on *Rosa*, evaluate the taxonomic value of selected morphological characters, and provide updated taxonomic treatments for the species examined. Two new species, *Phragmidium shennongjiaense* and *Phragmidium xinjiangense*, are described and illustrated. Emended descriptions and updated circumscriptions are also provided for 21 previously reported species.

## Materials and methods

### Specimen collection

Specimens of *Phragmidium* on *Rosa* were collected from multiple provinces in China, including Yunnan, Jiangxi, and Hubei, during field surveys from 2011 to 2019. Infected leaves and stems were pressed and dried following standard methods and deposited at the Mycological Herbarium of Beijing Forestry University (BJFC). In addition to newly collected materials, herbarium specimens were borrowed from the Herbarium Mycologicum Academiae Sinicae (HMAS) and the Mycological Herbarium of Ibaraki University (IBAR). All specimens were previously documented by Liu (2021), who included morphological descriptions, illustrations, and molecular sequences. In this study, these data were integrated into a multilocus phylogenetic framework to reassess species boundaries. GenBank accession numbers for sequences originally reported by Liu (2021) are provided in the species descriptions.

### Morphological observations

Morphological characteristics of the specimens, including urediniospores and teliospores, were originally described by Liu (2021) using light microscopy (Leica DM3000) and scanning electron microscopy (Hitachi S-3400N). For each specimen, at least 30 spores per spore stage were measured. These data were used here for species identification and comparative analyses. Detailed morphological descriptions and illustrations were documented by Liu (2021).

### DNA extraction, polymerase chain reaction, and sequencing

Molecular sequences used in this study were originally obtained and submitted to GenBank as part of a doctoral dissertation (Liu 2021). Detailed methods for DNA extraction, polymerase chain reaction (PCR) amplification, and sequencing followed the protocols described by Liu (2021). Briefly, DNA was extracted from 10–20 spores using the method of Tian et al. (2004), and the ITS and LSU regions were amplified with the primer pairs Rust2inv/ITS4rust (Aime 2006; Beenken et al. 2012) and LRust1R/LR3 (Vilgalys and Hester 1990), respectively.

### Phylogenetic analyses

Sequences were assembled using SeqMan v.7.1.0 (DNASTAR) and deposited in GenBank (Table 1). Reference sequences were retrieved from GenBank based on recent publications (Liu et al. 2018, 2019, 2020; Zhao et al. 2021; Sun et al. 2022). *Kuehneola uredinis* was selected as the outgroup (Aime 2006; Liu et al. 2020). Individual ITS and LSU alignments were generated using MAFFT v.7 (Katoh and Standley 2013), manually adjusted in MEGA v.7.0 (Kumar et al. 2016), and concatenated for combined analyses. Phylogenetic analyses were conducted using maximum parsimony (MP) in PAUP* v.4.0b10 (Swofford 2003) with 1000 bootstrap replicates, maximum likelihood (ML) in RAxML v.8.2.12 (Stamatakis 2014) on the CIPRES portal (Miller et al. 2010) with 1000 bootstrap replicates under the GTRGAMMA model, and Bayesian inference (BI) in MrBayes v.3.2.6 (Ronquist et al. 2012). For BI, the best-fit model was estimated with MrModeltest v.2.3 (Nylander et al. 2004); two Markov chain Monte Carlo (MCMC) runs were performed for 10 million generations, with sampling every 1000 generations and the first 25% discarded as burn-in. Trees were visualized in FigTree v.1.3.1 (Rambaut and Drummond 2010) and edited in Adobe Illustrator CS6 (Adobe Inc., USA).

**Table 1.** Collections used in phylogenetic analysis, locality, host, GenBank number and origin.

**Taxa**	**Accession number**	**GenBank accession**		**Host**	**Locality**	**References**
		**LSU**	**ITS**			
* Kuehneola uredinis*	BPI879274	GU058013	GU058013	* Rubus * sp.	USA	Dixon et al. 2010
* Phragmidium altaicum*	BJFCR03338	MK049169	MK049167	* Rosa canina*	Uzbekistan	Liu et al. 2019
	BJFCR03217^*^	MH285379	MH285383	* Rosa albertii*	Xinjiang China	Liu et al. 2020
* P. andersoni*	HMAS53231	MG669120	-	* Potentilla fruticosa*	Sinkiang, China	Liu et al. 2018
* P. barclayi*	HMAS67281	MG669117	-	* Rubus austroXizanganus*	Xizang, China	Liu et al. 2018
* P. barnardii*	HGUP21035	OL684839	OL684828	* Rubus parvifolius*	Guizhou, China	Sun et al. 2022
	BRIP56945	KT199402	-	* Rubus parvifolius*	South Africa	McTaggart et al. 2016
* P. biloculare*	BPI881121	JF907670	-	* Potentilla flabellifolia*	USA	Yun et al. 2011
* P. butleri*	HMAS67841	MG669118	-	* Rosa macrophylla*	Xizang, China	Liu et al. 2018
* P. bulbosum*	HMAS52930	-	MW729552	* Rubus caesius*	Xinjiang China	Direct Submission
* P. carbonarium*	CFSZ558	MW729481	-	* Sanguisorba officinalis*	Inner Mongolia, China	Direct Submission
	IBAR8722	MW729480	-	* Sanguisorba officinalis*	Yamagata, Japan	Direct Submission
* P. chayuensis*	BJFCR02532*	MG669112	-	* Rosa duplicata*	Xizang, China	Liu et al. 2018
	BJFCR03014	MG669113	-	* Rosa duplicata*	Xizang, China	Liu et al. 2018
* P. cibanum*	BJFCR02528*	MG669110	MH128370	* Rubus niveus*	Xizang, China	Liu et al. 2018
	BJFCR03012	MG669111	MH128371	* Rubus niveus*	Xizang, China	Liu et al. 2018
* P. cinnamomeum*	BJFCR03674	MW729486	MW729558	* Rosa sweginzowii*	Qinghai, China	Direct Submission
	BJFCR03678	MW729487	MW729559	* Rosa sweginzowii*	Qinghai, China	Direct Submission
* P. coreanicola*	GMB40101*	PQ456447	PQ472133	* Rubus coreanus*	Guizhou, China	Zhang et al. 2025
* P. coreanum*	BJFCR03867	MW729495	MW729567	* Rubus * sp.	Henan, China	Direct Submission
	BJFCR03868	MW729496	MW729568	* Rubus * sp.	Henan, China	Direct Submission
* P. duchesneae-indica*	HGUP21031	OL684835	OL684824	* Potentilla indica*	Guizhou, China	Sun et al. 2022
	HGUP21032	OL684836	OL684825	* Potentilla indica*	Guizhou, China	Sun et al. 2022
* P. fragariae*	WM 1317	AF426217	-	* Potentilla sterilis*	Europe	Maier et al. 2003
* P. fructigenum*	HMUT100472	KU059168	-	* Rosa glomerata*	Guangdong, China	Unpublished
* P. fusiforme*	T10	AJ715522	-	* Rosa pendulina*	Switzerland	Ritz et al. 2005
* P. griseum*	BJFCR03449	MN264730	MN264712	* Rubus crataegifolius*	Beijing, China	Liu et al. 2020
	BJFCR03451	MN264731	MN264713	* Rubus crataegifolius*	Beijing, China	Liu et al. 2020
	HMAS56906	MG669115	-	* Rubus crataegifolius*	Beijing, China	Liu et al. 2018
* P. handelii*	BJFCR01030	KP407631	-	* Rosa webbiana*	Gansu, China	Yang et al. 2015
	BJFCR01442	MW729500	MW729573	* Rosa tibetica*	Gansu, China	Direct Submission
	BJFCR01485	MW729501	MW729574	* Rosa * sp.	Gansu, China	Direct Submission
* P. himalayanum*	LAH37002	MW603008	-	* Rosa webbiana*	Pakistan	Afshan et al. 2010
	LAH37004	MW622041	-	* Rosa webbiana*	Pakistan	Afshan et al. 2010
* P. ivesiae*	BPI 877968	JF907673	-	* Potentilla gracilis*	USA	Yun et al. 2011
	BPI 863637	JF907672	-	* Potentilla gracilis*	USA	Yun et al. 2011
* P. japonicum*	HMAS41585	MN264734	MN264716	* Rosa laevigata*	Fujian, China	Liu et al. 2020
	IBAR8174	MN848143	MN882389	* Rosa luciae*	Ibaraki, Japan	Liu et al. 2020
* P. jiangxiense*	BJFCR03452	MN264732	MN264714	* Rosa laevigata*	Jiangxi, China	Liu et al. 2020
	BJFCR03453*	MN264733	MN264715	* Rosa laevigata*	Jiangxi, China	Liu et al. 2020
* P. kamtschatkae*	BJFCR03345 (TASMYG1085)	MW729504	MW729577	* Rosa ecae*	Uzbekistan, Jizzakh	Direct Submission
	BJFCR03344 (TASMYG1084)	MW729505	MW729578	* Rosa ecae*	Uzbekistan, Jizzakh	Direct Submission
* P. kanas*	HMAS24811	MK518678	MK518980	* Rosa fedtschenkoana*	Xinjiang, China	Zhao et al. 2021
	HMAS350018	MK518748	-	* Rosa fedtschenkoana*	Xinjiang, China	Zhao et al. 2021
* P. leucoaecium*	BJFCR02116	MN264736	MN264718	* Rosa * sp.	Yunnan, China	Liu et al. 2020
	BJFCR02118*	MN264737	MN264719	* Rosa * sp.	Yunnan, China	Liu et al. 2020
* P. mexicanum*	BPI843961	JF907660	-	* Potentilla indica*	USA	Yun et al. 2011
	BPI843829	JF907659	-	* Potentilla indica*	USA	Yun et al. 2011
* P. montivagum*	HMAS67176	KU059173	-	* Rosa davurica*	Inner Mongolia, China	Unpublished
* P. mucronatum*	BJFCR03455	MN264740	MN264722	* Rosa rugosa*	Beijing, China	Liu et al. 2020
	BJFCR03456	MN264741	MN264723	* Rosa rugosa*	Beijing, China	Liu et al. 2020
	BJFCR03557	MW729506	MW729579	* Rosa rugosa*	Beijing, China	Direct Submission
	BJFCR03558	MW729507	MW729580	* Rosa rugosa*	Beijing, China	Direct Submission
* P. nambuanum*	HMAS67221	-	MW729581	* Rubus crataegifolius*	Gansu, China	Direct Submission
* P. octoloculare*	HMAS140416	MG669119	MH128376	* Rubus biflorus*	Xizang, China	Liu et al. 2018
* P. parvifolius*	GMB4054*	PQ456454	PQ472140	* Rubus parvifolius*	Guizhou, China	Zhang et al. 2025
* P. pauciloculare*	HMAS140468	-	MW729583	* Rubus innominatus*	Hunan, China	Direct Submission
* P. potentillae*	HMAS53236	MG669114	-	* Potentilla virgata*	Sinkiang, China	Liu et al. 2018
	BJFCR00961	MN264738	MN264720	* Potentilla chinensis*	Qinghai, China	Liu et al. 2020
* P. potentillae-canadensis*	BPI877886	JF907667	-	* Potentilla * sp.	USA	Yun et al. 2011
	BPI877885	JF907668	-	* Potentilla canadensis*	USA	Yun et al. 2011
* P. potentillae-freynianae*	HGUP21033*	OL684837	OL684826	* Potentilla freyniana*	Guizhou, China	Sun et al. 2022
* P. punjabense*	BA65A*	KX358854	-	* Rosa brunonii*	Pakistan	Ali et al. 2017
	BA65B	KX358855	-	* Rosa brunonii*	Pakistan	Ali et al. 2017
* P. rosae-californicae*	MVAP0000154	MK045315	MK045315	* Rosa californica*	California, USA	Unpublished
* P. rosae-davuricae*	BJFCR00670	MW729511	MW729587	* Rosa oxyacantha*	Qinghai, China	Direct Submission
	BJFCR00651	MW729512	MW729588	* Rosa oxyacantha*	Qinghai, China	Direct Submission
* P. rosae-laevigatae*	HGUP21036*	OL684840	OL684829	* Rosa laevigata*	Guizhou, China	Sun et al. 2022
	HGUP21037	OL684841	OL684830	* Rosa laevigata*	Guizhou, China	Sun et al. 2022
* P. rosae-multiflorae*	HMAS71053	KU059174	-	* Rosa multiflora*	Shanxi, China	Unpublished
	HMAS94924	KU059175	-	* Rosa multiflora*	Zhejiang, China	Unpublished
	BJFCR03454	MN264739	MN264721	* Rosa multiflora*	Jiangxi, China	Liu et al. 2020
* P. rosae-roxburghii*	HGUP21025*	OL684831	OL684818	* Rosa roxburghii*	Guizhou, China	Sun et al. 2022
	HGUP21026	OL684832	OL684819	* Rosa roxburghii*	Guizhou, China	Sun et al. 2022
* P. rubi-corean*	HGUP21029*	OL684833	OL684822	* Rubus coreanus*	Guizhou, China	Sun et al. 2022
	HGUP21030	OL684834	OL684823	* Rubus coreanus*	Guizhou, China	Sun et al. 2022
* P. rubi-idaei*	WM 1024	AF426215	-	* Rubus idaeus*	Europe	Maier et al. 2003
	BRIP 59372	MW147044	-	* Rubus idaeus*	Australia	Aime and McTaggart 2020
* P. rubi-oldhami*	HMAS64306	MG669116	-	* Rubus pungens*	Sichuan, China	Liu et al. 2018
* P. rubi-thunbergii*	BJFCR03821	MW729521	MW729597	* Rubus corchorifolius*	Henan, China	Direct Submission
	BJFCR03822	MW729522	MW729598	* Rubus corchorifolius*	Henan, China	Direct Submission
* Phragmidium * sp.	HMAS41561	MN264735	MN264717	* Rosa multiflora*	Fujian, China	Liu et al. 2020
* P. sanguisorbae*	BPI 872232	JF907674	-	* Sanguisorba minor*	Germany	Yun et al. 2011
	HML957	AF426216	-	* Sanguisorba minor*	-	Maier et al. 2003
	BJFCR03347 (TASM-YG1087)	MW729523	MW729599	* Sanguisorba minor*	Jizzakh, Uzbekistan	Direct Submission
	BJFCR03348 (TASM-YG1088)	MW729524	MW729600	* Sanguisorba minor*	Jizzakh, Uzbekistan	Direct Submission
*** P. shennongjiaense***	BJFCR03275*	**MW729526**	**MW729602**	*** Rosa omeiensis***	**Hubei, China**	**This study**
	**BJFCR03696**	**MW729528**	**MW729604**	*** Rosa omeiensis***	**Qinghai, China**	**This study**
* P. tormentillae*	BPI843392	DQ354553	-	* Potentilla * sp.	USA	Yun et al. 2011
	BPI877888	JF907669	-	* Potentilla simplex*	USA	Yun et al. 2011
* P. tuberculatum*	BJFCR00951	MH285382	-	* Rosa * sp.	Qinghai, China	Unpublished
	BJFCR00959	KP407636	-	* Rosa * sp.	Qinghai, China	Yang et al. 2015
	BPI877978	KJ841919	-	* Rosa * sp.	USA	Wilson and Aime 2014
	BPI843677	KJ841921	-	* Rosa * sp.	Argentina	Wilson and Aime 2014
* P. violaceum*	MCA2782	DQ142909	DQ142909	* Rubus * sp.	France	Osterbauer et al. 2005
	BPI871510	DQ142910	DQ142910	* Rubus * sp.	USA	Osterbauer et al. 2005
	BJFCR03564	MN264742	MN264724	* Rubus * sp.	New Zealand	Liu et al. 2020
* P. warburgianum*	BJFCR03458 (IBAR7744)	MN264744	MN264726	* Rosa bracteata*	Japan	Liu et al. 2020
	BJFCR03459 (IBAR10130)	MN264745	MN264727	* Rosa bracteata*	Japan	Liu et al. 2020
*** P. xinjiangense***	BJFCR03250*	**MW729541**	**MW729619**	*** Rosa laxa***	**Xinjiang, China**	**This study**
	**BJFCR03214**	**MW729537**	**MW729615**	*** Rosa laxa***	**Xinjiang, China**	**This study**
* P. yangiae*	BJFCR00338*	MN264743	MN264725	* Rosa lichiangensis*	Yunnan China	Liu et al.2020
* P. zangdongii*	BJFCR02447*	MG669108	MH128372	* Rosa tibetica*	Xizang, China	Liu et al. 2018
	BJFCR03013	MG669109	MH128373	* Rosa tibetica*	Xizang, China	Liu et al. 2018
* P. zhouquensis*	BJFCR01516*	MN264746	MN264728	* Rosa omeiensis*	Yunnan China	Liu et al. 2020
	BJFCR01529	MN264747	MN264729	* Rosa omeiensis*	Yunnan China	Liu et al. 2020

*: type specimens or strains.

### Terminology

To describe life cycle stages, this study employs the ontogenic system of terminology (Hiratsuka 1973; Cummins and Hiratsuka 2003).

Sori preceded by or associated with spermogonia in the life cycle are recognized as the aecial stage. Aecia have a unique infection and developmental habit: they occur in aggregates on locally hypertrophied lesions on leaves, shoots, and even fruits. Severe spermogonial–aecial infection often causes malformation of entire shoots. When the sori show this type of developmental habit, they are identified as aecia regardless of whether spermogonia precede or are associated with their development. In macrocyclic species, uredinia and telia are scattered, although their density may be high and the sori may cover almost the entire leaf.

When only telia, with or without spermogonia, are found and the telial infection habit mimics the spermogonial–aecial infection habit, the fungus is considered to have a microcyclic life cycle.

For a different view of the rust life cycle and descriptive terminology, see Laundon (1967) and Holm (1987).

## Results

### Phylogenetic analyses

**Figure 1.**
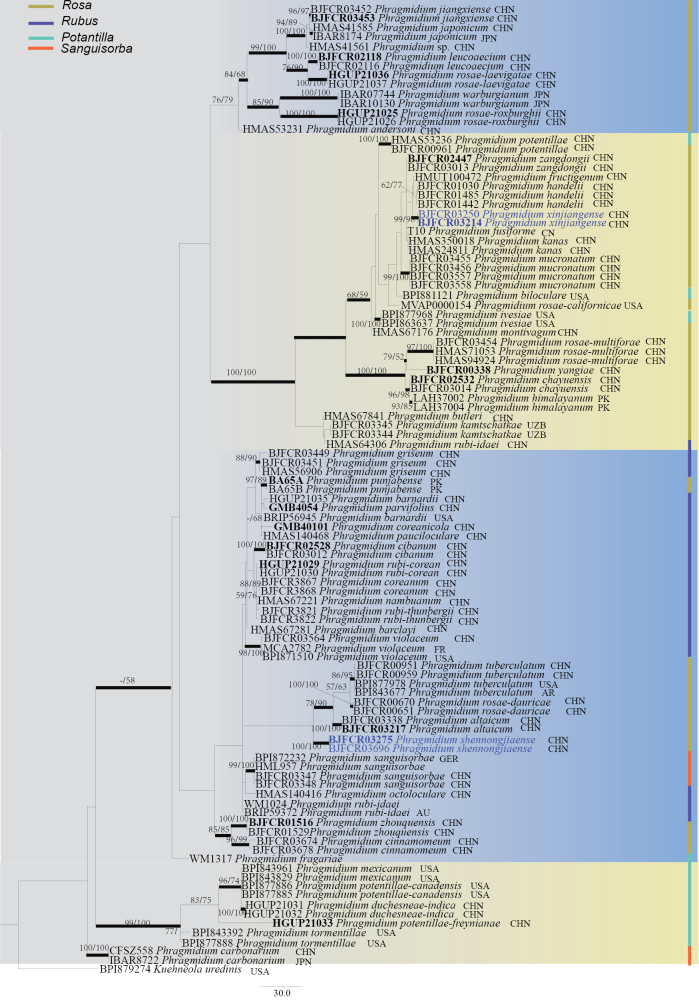
Phylogram based on the combined LSU and ITS sequences, constructed using maximum parsimony, maximum likelihood, and Bayesian inference. Bootstrap values > 50% are indicated above the branches. Thickened branches indicate PP > 0.90 from the Bayesian inference. Scale bar: 30.0 nucleotide substitutions. The newly discovered species are highlighted in bold and distinguished by blue. Type specimens are shown in bold; hosts are indicated by differently colored lines. Specimen collection localities are abbreviated by country: USA (United States), CHN (China), JPN (Japan), PK (Pakistan), UZB (Uzbekistan), AR (Argentina), AUS (Australia), and FR (France).

The combined ITS+LSU alignment comprised 1107 characters, of which 631 were constant, 79 were parsimony-uninformative, and 397 were parsimony-informative. Maximum parsimony analysis yielded 5000 equally parsimonious trees (TL = 1184, CI = 0.601, RI = 0.907, RC = 0.545), one of which is shown in Fig. 1. Phylogenetic trees generated from the ML and BI analyses were largely congruent with the MP tree. Phylogenetic analyses revealed that the specimens examined in this study formed 22 distinct, well-supported lineages corresponding to 23 species. Among these, 21 represent previously described species, and two are described as new species (*P. shennongjiaense* and *P. xinjiangense*). Bootstrap support values (≥ 50%) and Bayesian posterior probabilities (≥ 0.95) are indicated at the nodes.

### Taxonomy

#### Phragmidium shennongjiaense

Taxon classificationFungiPuccinialesPhragmidiaceae

Y.M. Liang, Ning Jiang & Yun Liu sp. nov.

CD7D4770-3F45-5567-8967-49CAA6E30700

862960

Fig. 2

##### Etymology.

The epithet “*shennongjiaense*” named from Shennongjia, the geographic site of the collection of this species.

##### Type.

China, • Hubei, Shennongyuan, on *Rosa omeiensis* Rolfe, 17 Aug. 2017, Qin Yang, Zhuo Du (holotype BJFC–R03275).

##### Description.

Spermogonia and aecia not seen. Uredinia hypophyllous, orange-yellow, scattered or clustered, rounded, ca. 0.2 mm diam.; paraphyses peripheral, clavate, incurved, hyaline, 34–55 × 9–16 μm; urediniospores pedicellate, subglobose to globose, 15–20 μm diam, wall 1.5–2.5 μm thick, with echinulae arising from a plaque-like base (plaque-echinulate type). Telia hypophyllous, pulverulent, black, scattered or clustered, rounded, 0.1–0.4 mm diam; teliospores cylindrical, light brown to dark brown, 6–9-celled (occasionally five), 73–91 × 24–29 μm, wall 1–2 μm thick, coarsely verrucose, apical papillae 3–12 μm long; pedicels 79–97 × 15–20 μm, swollen at lower 1/2 to 1/4 part.

##### Additional specimens examined.

China, • Qinghai, Haibei Xizangan Autonomous Prefecture, Haibei Xizangan Autonomous Prefecture, Menyuan Shierpan, on *Rosa omeiensis* Rolfe., 7 Jul. 2019, Yingmei Liang, Chengming Tian, Yun Liu, Cheng Peng (BJFC-R03675); • Qinghai, Haibei Xizangan Autonomous Prefecture, Qilian County, Jiulian Valley, 13 Jul. 2019, Yingmei Liang, Bin Cao (BJFC-R03696, BJFC-R03700); on *Rosa* sp., Gansu, Wuwei City, Tianzhu County, 19 Aug. 2018, Bin Cao (BJFC–R03553).

**Figure 2.**
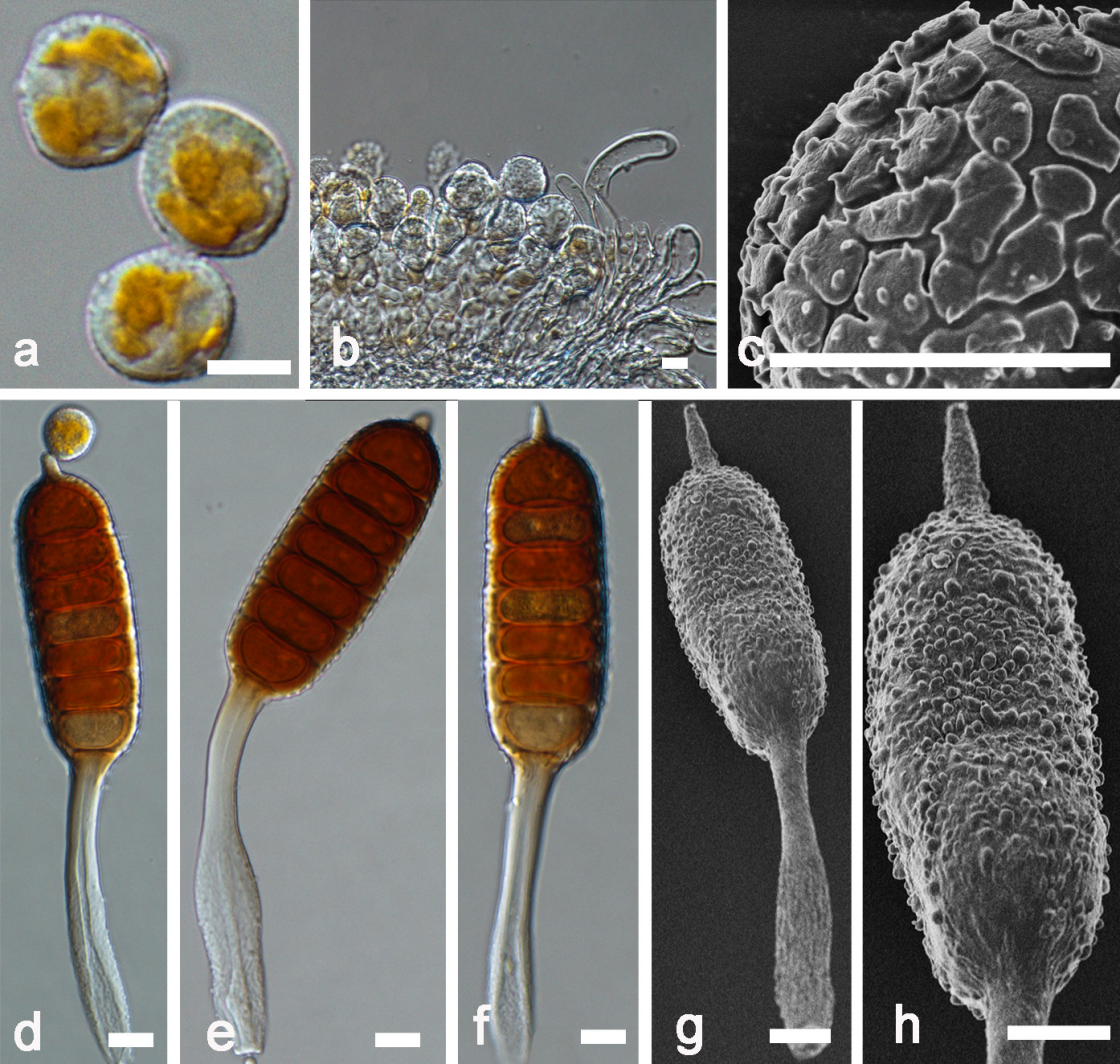
*
Phragmidium shennongjiaense* (BJFC-R03275). **a**. Urediniospores (LM); **b, c**. Urediniospores (SEM); **d–f**. Teliospores (LM); **g, h**. Teliospores (SEM). Scale bars: 10 μm (**a–h**). From Liu (2021).

##### GenBank numbers.

ITS: MW729601 (BJFC-R03553), MW729602 (BJFC-R03275), MW729603 (BJFC-R03675), MW729604 (BJFC-R03696), MW729605 (BJFC-R03700); LSU: MW729525 (BJFC-R03553), MW729526 (BJFC-R03275), MW729527 (BJFC-R03675), MW729528 (BJFC-R03696), MW729529 (BJFC-R03700).

##### Notes.

The combination of 6–9-celled teliospores (73–91 × 24–29 μm) with coarsely verrucose walls and pedicellate urediniospores bearing echinulae on a plaque-like base (plaque-echinulate type) distinguishes this species from its relatives (Fig. 1). Morphologically, it resembles *P. montivagum* (Wei 1988; Zhuang et al. 2012) and *P. chayuensis* (Liu et al. 2018; Liu 2021) but differs in having plaque-echinulate urediniospores (vs. densely echinulate urediniospores) and longer apical papillae (3–12 μm vs. up to 5 μm). In the phylogenetic tree, *P. shennongjiaense* forms a distinct clade sister to *P. rubi*-*idaei* with strong support (ML/MP/BI = 100/100/1) (Fig. 1), confirming its status as a distinct species.

#### Phragmidium xinjiangense

Taxon classificationFungiPuccinialesPhragmidiaceae

Y.M. Liang, Ning Jiang & Yun Liu sp. nov.

87781CFB-0B34-58AA-8F2B-A61FAE10C95E

862961

Fig. 3

##### Etymology.

The specific epithet “*xinjiangense*” refers to Xinjiang, the geographic locality where the type specimen was collected.

##### Type.

China, • Xinjiang, Bortala Mongol Autonomous Prefecture, Bole City, Haritureg National Forest Park, on *Rosa laxa* Retz, 18 Jul. 2017, Yingmei Liang and Yun Liu (holotype BJFC-R03250).

##### Description.

Spermogonia and aecia not seen. Uredinia hypophyllous, orange-yellow, scattered or clustered, rounded, 0.1–0.2 mm diam; paraphyses peripheral, incurved, hyaline, 37–58 × 13–17 μm; urediniospores ellipsoid to globose, hyaline to orange-yellow, 16–23 × 16–19 μm, wall 1–2 μm thick, densely echinulate. Telia hypophyllous, pulverulent, black, scattered or clustered, sometimes mixed with uredinia, rounded, 0.4–0.6 mm diam.; teliospores cylindrical to fusiform, dark brown, 7–10-celled (occasionally six or 11), 47–72 × 19–24 μm, wall ca. 1 μm thick, coarsely verrucose, apical papillae 2–7 μm long; pedicels 71–94 × 11–14 μm, weakly swollen at lower half.

##### Additional specimens examined.

China, • Xinjiang, Altay Prefecture, Altay City, Xiaodonggou, on *Rosa rugosa* Thunb., 12 Jul. 2017, Yingmei Liang and Yun Liu (BJFC-R03213, BJFC-R03214, BJFC-R03220, BJFC-R03221); on *Rosa xanthina* Lindl., 13 Jul. 2017, Yingmei Liang and Yun Liu (BJFC-R03224); • Xizang, Nyingchi City, Bayi District, Lulang Town, 25 Jul. 2016, Yingmei Liang and Jing Cao (BJFC-R02456).

##### GenBank numbers.

ITS: MW729614 (BJFC-R03213), MW729615 (BJFC-R03214), MW729616 (BJFC-R03220), (BJFC-R03221), MW729618 (BJFC-R03224), MW729619 (BJFC-R03250); LSU: MW729536 (BJFC-R03213), MW729537(BJFC-R03214), MW729538 (BJFC-R03220), MW729539 (BJFC-R03221), MW729540 (BJFC-R03224), MW729541 (BJFC-R03250).

##### Notes.

**Figure 3.**
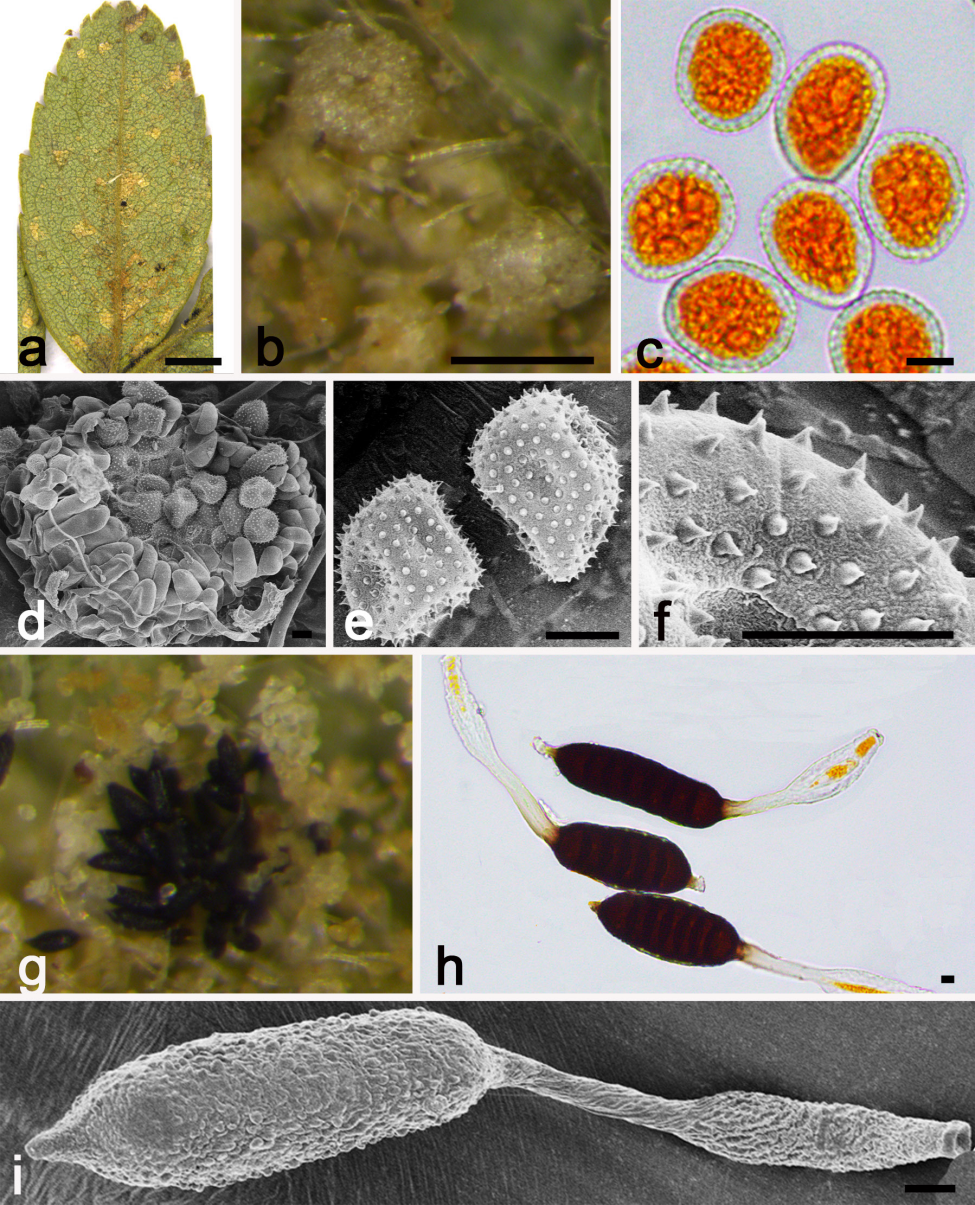
*
Phragmidium xinjiangense* (BJFC-R03250). **a, g**. Telia and uredinia; **b, d**. Uredinia; **c**. Urediniospores (LM); **e, f**. Urediniospores (SEM); **h**. Teliospores (LM); **i**. Teliospore (SEM). Scale bars: 1 mm (**a, b**); 10 μm (**c–f, h, i**). From Liu (2021).

This fungus is morphologically characterized by 7–10-celled teliospores (47–72 × 19–24 μm) with coarsely verrucose walls and apical papillae 2–7 μm long and densely echinulate urediniospores (Fig. 3). It differs from the morphologically similar *P. montivagum* (Wei 1988; Zhuang et al. 2012) and *P. chayuensis* (Liu et al. 2018; Liu 2021) in having shorter teliospores (47–72 μm vs. 69–89 μm in *P. montivagum* and 67–87 μm in *P. chayuensis*) and more teliospore cells (7–10 vs. 7–9 in both species). Phylogenetically, *P. xinjiangense* forms a distinct clade with strong support (ML/MP/BI = 100/100/1) (Fig. 1), confirming its status as a distinct species.

#### Phragmidium yangiae

Taxon classificationFungiPuccinialesPhragmidiaceae

Berndt & B. Ali, in Ali, Sohail, Mumtaz and Berndt, Beih. Nova Hedwigia: 9 (Ali et al. 2017) as “ yangii”

47B60C53-B716-5E74-8B40-60D73A41F3DA

##### Synonyms.

*Phragmidium longissima* Y.M. Liang & T. Yang, in Yang, Chang, Cao, Tian, Zhao & Liang, Phytotaxa 217(2): 186.

##### Description.

Spermogonia and aecia not seen. Uredinia hypophyllous, 0.05–0.2 mm diam.; urediniospores 20–26 × 18–21 μm, densely echinulate. Telia hypophyllous, black; teliospores 9–11-celled, 85–122 × 21–30 μm, coarsely verrucose, apical papillae 2.5–5.5 μm long; pedicels 65–111 × 11–17 μm, swollen at base.

##### Specimens examined.

• Yunnan, Nujiang Lisu Autonomous Prefecture, Lanping Bai and Pumi Autonomous County, Luoguqing Primary Forest, on *Rosa lichiangensis* Yu et Ku., 19 Sept. 2011, Ting Yang (Holotype BJFC-R00338).

##### GenBank numbers.

ITS: MN264725 (BJFC-R00338); LSU: MN264743 (BJFC-R00338).

##### Notes.

The species is characterized by 9–11-celled teliospores (85–122 × 21–30 μm) and densely echinulate urediniospores. Phylogenetically, it forms a well-supported clade within *Phragmidium* (Fig. 1), distinct from other species on *Rosa*.

#### Phragmidium altaicum

Taxon classificationFungiPuccinialesPhragmidiaceae

Y.M. Liang & Yun. Liu, Phytotaxa 423 (3): 187–194. 2019

ABFE2729-537C-57E4-A593-186BAA3F4905

##### Description.

Spermogonia and aecia not seen. Uredinia hypophyllous, yellow, 0.5–1 mm diam.; urediniospores 17–22 × 15–19 μm, wall ca. 1 μm thick, echinulate. Telia hypophyllous, black, 0.3–0.7 mm diam.; teliospores 4–7-celled, 30–75 × 23–28 μm, wall 2.5–5 μm thick, coarsely verrucose, apical papillae 4–10 μm long; pedicels 62–92 × 14–20 μm, swollen at base.

##### Specimens examined.

China, • Xinjiang, Tacheng City, Jimunai County, Tusti Valley, on *R. beggeriana* Schrenk, 15 Jul. 2017, Chengming Tian, Yingmei Liang, Yun Liu (BJFC-R03246); • Xinjiang, Altay City, on *Rosa albertii* Regel, 12 July 2017, Yingmei Liang and Yun Liu (holotype BJFC-R03217); • Xinjiang, Bortala Mongol Autonomous Prefecture, Bole City, Junzhureke Village, on *R. laxa* Retz, 16 Jul. 2017, Chengming Tian, Yingmei Liang, Yun Liu (BJFC-R03247); Uzbekistan, • Zomin National Park, on *Rosa maracandica* Bunge., 26 May 2018, Y. Gafforov (TASM–YG1073=BJFC-R03338); Uzbekistan, • Zomin National Park, on *Rosa canina* L., 28 May 2018, Y. Gafforov (TASM-YG1069=BJFC-R03336). For a complete list of all specimens examined, see Suppl. material 1.

##### GenBank numbers.

ITS: MH285383 (BJFC-R03217), MH285379 (BJFC-R03338), MK049168 (BJFC-R03336), MH285384 (BJFC-R03246), MH285385 (BJFC-R03247); LSU: MK049168 (BJFC-R03217), MK049169 (BJFC-R03338), MK049170 (BJFC-R03336), MH285380 (BJFC-R03246), MH285381 (BJFC-R03247).

##### Notes.

Originally described from Xinjiang on *Rosa albertii* (Liu et al. 2019), the taxonomic circumscription of *Phragmidium altaicum* is emended to encompass 4–7-celled teliospores (30–75 × 23–28 μm) with robust verrucae, echinulate type 4 aeciospores (Wahyuno et al. 2002) with candle-echinulate ornamentation (Liu 2021), and urediniospores possessing mushroom-shaped basal spines. Although phylogenetically sister to *P. tuberculatum* and *P. rosae*-*davuricae* (Fig. 1), *P. altaicum* is clearly delimited by this unique combination of aeciospore and urediniospore ultrastructure.

#### Phragmidium butleri

Taxon classificationFungiPuccinialesPhragmidiaceae

Syd, Ann. Mycol 5: 501. 1907

50D7B225-A363-5444-8978-F579E4EBFF11

##### Description.

Spermogonia not seen. Mixed occurrence of aeciospores and urediniospores, occurring on the abaxial leaf surface. Aeciospores are circular, pyriform, or irregular in shape, measuring 15–27 × 17–22 μm. Their surface is ornamented with 1–5 spines borne on raised, tray-like basal structures (plaque-spine type PE). Uredinia hypophyllous, 0.5–1 mm diam.; urediniospores 17–30 × 15–22 μm, densely echinulate. Telia hypophyllous, black, 0.5–1 mm diam.; teliospores 5–8-celled, 70–105 × 25–35 μm, coarsely verrucose, apical papillae up to 12 μm long; pedicels 90–125 × 15–25 μm, swollen at base.

##### Specimens examined.

China, • Xizang, Shigatse, Gyirong County, on *Rosa macrophylla* Lindl, 9 Sept. 1990, Jianyun Zhuang (HMAS67841). For a complete list of all specimens examined, see Suppl. material 1.

##### GenBank numbers.

ITS: MW729553 (HMAS67841); LSU: MG669118 (HMAS67841).

##### Notes.

First described from India on *Rosa macrophylla* (Sydow et al. 1906), this species is characterized by 5–8-celled teliospores (70–105 × 25–35 μm) with coarsely verrucose walls and apical papillae up to 12 μm long (Liu 2021). Although morphologically similar to *P. tuberculatum* and *P. rosae-davuricae*, it occupies a separate phylogenetic lineage in the molecular tree (Fig. 1), supporting its recognition as a distinct species.

#### Phragmidium chayuensis

Taxon classificationFungiPuccinialesPhragmidiaceae

Y.M. Liang & Yun. Liu, Mycological Progress 17(8): 967–988. 2018

F4995E02-A3CC-55B4-8ED2-A10F29FCAA0A

##### Description.

Spermogonia and aecia not seen. Uredinia hypophyllous, 0.1–0.3 mm diam.; urediniospores 19–23 × 17–22 μm, densely echinulate. Telia hypophyllous, black, 0.2–0.5 mm diam.; teliospores 7–9-celled, 67–87 × 22–30 μm, coarsely verrucose, apical papillae absent or up to 5 μm long; pedicels 75–103 × 15–18 μm, swollen at base.

##### Specimens examined.

China, • Xizang, Nyingchi City, Zayü County, Zhuwagen Town, on *Rosa duplicata* T. T. Yu & T. C.Ku, 30 Jul. 2016, Yingmei Liang and Jing Cao (BJFC-R02532 (holotype), BJFC-R03014 (isotype)); • Gansu, Changde City, Wuling District, Taiyangshan Forest Park, on *Rosa rubus* Lévl. et Vant., 18 Jul. 2019, Yingmei Liang, Chengming Tian, Yun Liu, Cheng Peng (BJFC-R03716, BJFC-R03717, BJFC-R03718). For a complete list of all specimens examined, see Suppl. material 1.

##### GenBank numbers.

ITS: MH128374 (BJFC-R02532), MH128375 (BJFC-R03014), MW729554 (BJFC-R03716), MW729555 (BJFC-R03717), MW729556 (BJFC-R03718); LSU: MG669112 (BJFC-R02532), MG669113 (BJFC-R03014), MW729482 (BJFC-R03716), MW729483 (BJFC-R03717), MW729484 (BJFC-R03718).

##### Notes.

This species was first discovered in Xizang, China, on *Rosa duplicata* (Liu et al. 2018). Its 7–9-celled teliospores (67–87 × 22–30 μm), with coarsely verrucose walls and short or absent apical papillae (up to 5 μm), distinguish it from morphologically similar species such as *P. hashiokae* (100–150 × 25–36 μm), *P. montivagum* (apical papillae up to 13 μm), and *P. rosae-multiflorae* (Liu 2021). Phylogenetically, it forms a well-supported clade sister to *P. rosae-multiflorae* (Fig. 1) and represents the first *Phragmidium* species reported on *R. duplicata*.

#### Phragmidium cinnamomeum

Taxon classificationFungiPuccinialesPhragmidiaceae

Durrieu, Cryptog. Mycol. 1(1): 51. 1980

335681A6-E2CA-551C-A4A9-3C5A39E12DFA

##### Description.

Spermogonia and aecia not seen. Uredinia hypophyllous, light yellow, 0.3–1.5 mm diam.; urediniospores ellipsoid to globose, 12–17 × 15–19 μm, wall ca. 2 μm thick, echinulate. Telia hypophyllous, black, 0.3–1.5 mm diam.; teliospores 5–6-celled (occasionally four or eight), 58–67 × 24–29 μm, wall ca. 3 μm thick, coarsely verrucose, apical papillae 6–16 μm long; pedicels 42–115 × 6–14 μm, slightly swollen at base.

##### Specimens examined.

China, • Qinghai, Haibei Tibetan Autonomous Prefecture, Haiyan County, Yuershan, 9 Jul. 2019, Yingmei Liang, Chengming Tian, Yun Liu, Cheng Peng (BJFC-R03689); • Qinghai, Haibei Tibetan Autonomous Prefecture, Menyuan Hui Autonomous County, Xianmi Forest Farm, 9 Jul. 2019, Yingmei Liang, Chengming Tian, Yun Liu, Cheng Peng (BJFC-R03695, BJFC-R03686); • Qinghai, Huangnan Tibetan Autonomous Prefecture, Zeku County, Maixiu Forest Farm Jiuliangou, 13 Jul. 2019, Yingmei Liang, Chengming Tian, Yun Liu, Cheng Peng (BJFC-R03698, BJFC-R03701, BJFC-R03706); • Qinghai, Haibei Tibetan Autonomous Prefecture, Menyuan Hui Autonomous County, Menyuan 12 Pan, on *R. sweginzowii* Koehne., 7 Jul. 2019, Yingmei Liang, Chengming Tian, Yun Liu, Cheng Peng (BJFC-R03674, BJFC-R03678). For a complete list of all specimens examined, see Suppl. material 1.

##### GenBank numbers.

ITS: MW729557 (BJFC-R02086), MW729558 (BJFC-R03674), MW729559 (BJFC-R03678), MW729560 (BJFC-R03686), MW729561 (BJFC-R03689), MW729562 (BJFC-R03695), MW729563 (BJFC-R03698), MW729564 (BJFC-R03701), MW729565 (BJFC-R03706); LSU: MW729485 (BJFC-R02086), MW729486 (BJFC-R03674), MW729487 (BJFC-R03678), MW729488 (BJFC-R03686), MW729489 (BJFC-R03689), MW729490 (BJFC-R03695), MW729491 (BJFC-R03698), MW729492 (BJFC-R03701), MW729493 (BJFC-R03706).

##### Notes.

Originally described from Nepal (Durrieu 1980), this species is common in China on *Rosa* species from Qinghai, Gansu, and Xizang (Liu 2021). It is characterized by 5–6-celled teliospores (58–67 × 24–29 μm) with coarsely verrucose walls and apical papillae 6–16 μm long. Although it resembles *P. tuberculatum* in teliospore cell number and urediniospore ornamentation, the chestnut-colored telia and teliospores (vs. black telia and dark brown teliospores in *P. tuberculatum*) provide a reliable morphological distinction. The phylogenetic tree places *P. cinnamomeum* as sister to *P. zhouquense* with strong support (Fig. 1).

#### Phragmidium fusiforme

Taxon classificationFungiPuccinialesPhragmidiaceae

J. Schröt., Abh. Schles. Ges. Vaterl. Kult., Abth. Naturw. Med. 48: 24. 1870

B169AE0C-FB3B-5208-9827-6765FD67CCA2

##### Description.

Spermogonia and aecia not seen. Uredinia hypophyllous, white, 0.1–0.6 mm diam.; urediniospores 15–25 × 16–20 μm, densely echinulate. Telia hypophyllous, black, 0.1–0.5 mm diam.; teliospores 11–13-celled, 93–125 × 17–28 μm, coarsely verrucose, apical papillae up to 13 μm long; pedicels 50–150 × 15–18 μm, slightly swollen at base.

##### Specimens examined.

China, • Gansu, Wenshan Zhuang and Miao Autonomous Prefecture, Maguan County, Ma Guan Mountain, on *Rosa sweginzowii* Koehne, 2 Sept. 2004, Ruili Ma (HMAS140421); • Gannan Tibetan Autonomous Prefecture, Diebe County, 11 Sept. 1992, Jianyun Zhuang (HMAS67193); Gansu, Zhouqu County, on *R. sertata* Rolfe, 4 Sept. 1992, Jianyun Zhuang (HMAS67193); • Xizang, Nyingchi City, Bomi County, on *R. moyesii* Hemsl. & E.H. Wilson, 26 Aug. 1983, Jianyun Zhuang (HMAS45219–HMAS45220); • Hubei, Shennongjia, on *R. corymbulosa* Rolfe, 2 Aug. 1984, Lin Guo (HMAS55451); • Xinjiang, on *R. acicularis* Lindl. Kanas Nature Reserve, 5 Sept. 2004, Jianyun Zhuang (HMAS172069).

##### Notes.

Among *Phragmidium* species on *Rosa*, this taxon is unique in having 11–13-celled teliospores (93–125 × 17–28 μm) that are nearly fusiform, tapering toward both ends (Liu 2021). It is one of the few species in the genus with more than 10 teliospore cells. Phylogenetic analysis places it in a well-supported clade distinct from other species (Fig. 1).

#### Phragmidium handelii

Taxon classificationFungiPuccinialesPhragmidiaceae

Petr., Meddn Göteb. Bot. Trädg. 17: 121. 1947

495EFBC6-4EEB-5606-8AF1-061E265CB222

##### Description.

Spermogonia, aecia, and uredinia not seen. Telia black, pulverulent, on fruits and branches, up to 2 cm diam.; teliospores 12–16-celled, 69–101 × 19–24 μm, coarsely verrucose, apical papillae 2–10 μm long; pedicels 75–132 × 10–16 μm, slightly swollen at base.

##### Specimens examined.

China, • Gansu, Longnan City, Huagou, on *Rosa corymbulosa* Rolfe, 17 Jul. 2019, Yingmei Liang, Chengming Tian, Yun Liu, Cheng Peng (BJFC-R03713); 4 May 2017, Bin Cao (BJFC-R02982); • Gansu, Lintan County, Yeli Guan Daling Mountain, on *R. webbiana* Wall. ex Royle, 15 Aug. 2014, Yingmei Liang, Chengming Tian, Bin Cao (BJFC-R01442); • Gansu, Gannan Tibetan Autonomous Prefecture, Diebu County, Lazikou Checkpoint, 18 Aug. 2014, Yingmei Liang, Chengming Tian, Bin Cao (BJFC-R01485). For a complete list of all specimens examined, see Suppl. material 1.

##### GenBank numbers.

ITS: MW729573 (BJFC-R01442), MW729574 (BJFC-R01485), MW729575 (BJFC-R02982), MW729576 (BJFC-R03713); LSU: MW729500 (BJFC-R01442), MW729501 (BJFC-R01485), MW729502 (BJFC-R02982), MW729503 (BJFC-R03713).

##### Notes.

Distinguished by its 12–16-celled teliospores (69–101 × 19–24 μm), this species possesses the highest number of teliospore cells recorded in the genus (Liu 2021). It forms large, crust-like telia on fruits and branches, often causing host deformities. In the phylogenetic tree, it occupies a well-supported clade distinct from other *Rosa*-associated species (Fig. 1).

#### Phragmidium japonicum

Taxon classificationFungiPuccinialesPhragmidiaceae

Dietel, Bot Jahrb 27:567. 1899

3CB13BE2-32A5-509D-AFD7-0AB77A79B964

##### Description.

Spermogonia, aecia, and uredinia not seen. Telia amphigenous, bright orange or reddish orange when fresh, 0.1–0.5 mm diam.; teliospores 2–5-celled, 30–100 μm long, smooth; pedicels less than 10 μm long.

##### Specimens examined.

China, • Fujian, Nanping City, Wuyi Mountain, on *Rosa laevigata* Michx, 8 Oct. 1980, Jianyun Zhuang (HMAS41585); Japan, • Ibaraki, on *R. luciae* Franchet & Rochebrune, 12 Aug. 1998, Y. Ono and K. Ishimiya (IBAR8174). For a complete list of all specimens examined, see Suppl. material 1.

##### GenBank numbers.

ITS: MN264716 (HMAS41585), MN882389 (IBAR8174); LSU: MN264734 (HMAS41585), MN848143 (IBAR8174).

##### Notes.

Originally described from Japan on *Rosa multiflora* (Dietel and Neger 1899), this species was later transferred to *Kuehneola* as *K. japonica* (Dietel 1912). Molecular phylogenetic analysis unequivocally places it within *Phragmidium* (Liu et al. 2020; Fig. 1), restoring its original generic placement. It is readily identified by its 2–5-celled teliospores (30–100 μm long) with smooth walls and very short pedicels (< 10 μm). Its microcyclic life cycle has been experimentally confirmed (Ono 2002).

#### Phragmidium jiangxiense

Taxon classificationFungiPuccinialesPhragmidiaceae

Y.M. Liang & Y. Liu, Mycologia 112(4): 747. 2020

D9315CC4-1324-591D-AE24-225D0E639B6B

##### Description.

Spermogonia and telia not seen. Aecia hypophyllous, orange, 0.2–0.5 mm diam.; aeciospores 13–22 × 12–17 μm, echinulate. Uredinia hypophyllous; urediniospores 15–23 × 11–18 μm, wall 0.5–2 μm thick, with stout spines basally immersed in wrinkled wall.

##### Specimens examined.

China, • Jiangxi, Ganzhou City, Chongyi County, Qiyun Mountain, on *Rosa laevigata* Michx., 20 Jul. 2018, Chengming Tian, Yingmei Liang, Yun Liu (BJFC-R03452, BJFC-R03453). For a complete list of all specimens examined, see Suppl. material 1.

##### GenBank numbers.

ITS: MN264714 (BJFC-R03452), MN264715 (BJFC-R03453); LSU: MN264732 (BJFC-R03452), MN264733 (BJFC-R03453).

##### Notes.

Described from Jiangxi, China, on *Rosa laevigata* (Liu et al. 2020), this species is immediately recognizable by its urediniospores with stout spines basally immersed in a wrinkled spore wall—a feature unique among *Phragmidium* species on *Rosa* (Liu 2021). Although closely related to *P. japonicum* (Fig. 1), this fungus differs in having a macrocyclic life cycle (vs. microcyclic) and wrinkled urediniospores (vs. smooth teliospores).

#### Phragmidium kamtschatkae

Taxon classificationFungiPuccinialesPhragmidiaceae

(F.W. Anderson) Arthur & Cummins, Mycologia 25(5): 401. 1933

32AC171A-0B28-52FF-B698-D9A3AB7A35CA

##### Description.

Spermogonia and uredinia not seen. Aecia on twigs, ca. 2 mm diam., orange-yellow; aeciospores 16–21 μm diam., hyaline, echinulate. Telia amphigenous, yellowish-brown; teliospores two-celled, 28–40 × 19–22 μm, sparsely verrucose, without apical papillae; pedicels atrophied.

##### Specimens examined.

Uzbekistan, • Jizzakh Region, Zaamin District, Zaamin National Park, on *Rosa ecae* Aitch., 26 May 2018, Y. Gafforov (BJFC-R03344, BJFC-R03345). For a complete list of all specimens examined, see Suppl. material 1.

##### GenBank numbers.

ITS: MW729577 (BJFC-R03345 (TASM-YG1085)), MW729578 (BJFC-R03344 (TASM-YG1084)); LSU: MW729504 (BJFC-R03345 (TASM-YG1085)), MW729505 (BJFC-R03344 (TASM-YG1084)).

##### Notes.

First described as *Puccinia kamtschatkae* and later transferred to *Phragmidium* (Arthur and Cummins 1933), the taxonomic placement of this species has been debated because of its 2-celled teliospores and atrophied pedicels (Zhuang et al. 2012). Phylogenetic analysis now confirms its placement within *Phragmidium* (Fig. 1). It is characterized by 2-celled teliospores (28–40 × 19–22 μm) with sparse verrucae and aeciospores with plaque-echinulate ornamentation.

#### Phragmidium leucoaecium

Taxon classificationFungiPuccinialesPhragmidiaceae

Y.M. Liang & Y. Liu, Mycologia 112(4): 747. 2020

B7BC7124-DF76-5EDD-AF6C-892375FC23E4

##### Description.

Uredinia and telia not seen. Spermogonia hypophyllous, type 2, 103–220 × 55–70 μm. Aecia hypophyllous, up to 12 mm long, white to yellowish, aparaphysate; aeciospores 24–40 × 12–22 μm, hyaline, with irregularly elongated verrucae toward the apex.

##### Specimens examined.

China, • Yunnan, Dali Bai Autonomous Prefecture, Binchuan County, Jizu Mountain, on *Rosa* sp., 22 Mar. 2015, Bin Cao and Qin Yang (BJFC-R02118); Yunnan, Kunming City, Panlong District, Back Hill of Yunnan Agricultural University, 16 Mar. 2015, Qin Yang (BJFC-R02116).

##### GenBank numbers.

ITS: MN264718 (BJFC-R02116), MN264719 (BJFC-R02118); LSU: MN264736 (BJFC-R02116), MN264737 (BJFC-R02118).

##### Notes.

Described from Yunnan, China, on *Rosa* sp. (Liu et al. 2020), this species possesses two unusual features: type 2 spermogonia (Group I, type 2) (Cummins and Hiratsuka 2003), which are rare in *Phragmidium*, and aeciospores with irregularly elongated verrucae interconnected by narrow ridges (echinulate-verrucose type). This combination of characters sets it apart from all other *Phragmidium* species on *Rosa*. Phylogenetic analysis places it in a well-supported clade distinct from its relatives (Fig. 1).

#### Phragmidium montivagum

Taxon classificationFungiPuccinialesPhragmidiaceae

Arthur, Torreya 9: 24. 1909

7A2DABAC-A001-5B90-A968-7238C3993250

##### Description.

Spermogonia and aecia not seen. Uredinia hypophyllous, 0.1–0.4 mm diam.; urediniospores 20–23 × 14–18 μm, densely echinulate. Telia hypophyllous, black, 0.1–0.5 mm; teliospores 7–9-celled, 69–89 × 28–35 μm, coarsely verrucose, apical papillae 8–11 μm long; pedicels 91–130 × 18–25 μm, swollen at base.

##### Specimens examined.

China, • Nei Mongol, Alshan, on *Rosa davurica* Pall., 15 Aug. 1911, Zhuang Jianyun (HMAS67176). For a complete list of all specimens examined, see Suppl. material 1.

##### GenBank numbers.

LSU: KU059173 (HMAS67176).

##### Notes.

This species is easily recognized by its teliospores, which are widest in the middle and taper toward both ends (subfusiform), measuring 69–89 × 28–35 μm and comprising 7–9 cells (Liu 2021). This distinctive shape distinguishes it from other species with similar teliospore cell numbers. Phylogenetic analysis supports its placement in a well-supported clade (Fig. 1).

#### Phragmidium mucronatum

Taxon classificationFungiPuccinialesPhragmidiaceae

(Pers.) Schltdl., Fl. berol. (Berlin) 2: 156. 1824

BFD19C46-1088-5633-B911-3A305B495FFF

##### Description.

Spermogonia and aecia not seen. Uredinia hypophyllous, yellow to orange, 0.5–3 mm in diameter or up to 3 mm long; urediniospores broadly ellipsoid to subglobose, 18–25 × 16–23 μm, densely to finely echinulate or echinulate-verruculose, with conspicuous germ pore caps. Telia hypophyllous, brown to chestnut-brown when young, becoming black at maturity; teliospores 6–10-celled, 73–133 × 26–35 μm, coarsely verrucose, with apical papillae 3–10 μm long; pedicels 78–180 × 10–24 μm, gradually thickened downward and swollen at the base. For detailed descriptions and illustrations, see Liu (2021).

##### Specimens examined.

China, • Beijing, Mentougou District, Mentougou Rose Garden, on *Rosa rugosa* Thun., 29 sep. 2019, Chengming Tian, Yingmei Liang and Yun Liu (BJFCR03455, BJFCR03456, BJFCR03557, BJFCR03558). For a complete list of all specimens examined, see Suppl. material 1.

##### GenBank numbers.

ITS: MN264722 (BJFCR03455), MN264723 (BJFCR03456), MW729579 (BJFCR03557), MW729580 (BJFCR03558); LSU: MN264740 (BJFCR03455), MN264741 (BJFCR03456), MW729506 (BJFCR03557), MW729507 (BJFCR03558).

##### Notes.

As the type species of the genus *Phragmidium*, this taxon holds special nomenclatural significance. It is distinguished by its urediniospores, which possess a distinct pore membrane—a feature not observed in other *Phragmidium* species on *Rosa*. The species is widespread globally and forms a well-supported clade in the phylogenetic tree (Fig. 1). The Chinese collections examined here are referred to *Phragmidium mucronatum* following the traditional morphological concept of the species. They agree with the original description and later taxonomic treatments in having urediniospores with conspicuous pore caps, which have long been regarded as a characteristic feature of *P. mucronatum* (Wilson and Henderson 1966). The teliospores are also mostly 6–8-celled, as described in previous accounts. However, the type specimen and topotypic European material were not available for examination in this study. Moreover, the available GenBank sequences identified as *P. mucronatum* were used only for comparison because some records lack sufficient voucher or morphological information. Further study of European material, preferably including topotypic collections, together with newly collected Chinese specimens, is needed to clarify the relationship between Chinese and European populations of *P. mucronatum*.

#### Phragmidium robustum

Taxon classificationFungiPuccinialesPhragmidiaceae

J.Y. Zhuang & S.X. Wei, Mycosystema 28(5): 625. 2009

88F86079-8914-55E8-9FBB-49EDE0F0B869

##### Description.

Spermogonia, aecia, and uredinia not seen. Telia hypophyllous, brown, 0.1–0.3 mm; teliospores 5–6-celled, 68–88 × 32–38 μm, coarsely verrucose, apical papillae 1–4 μm long; pedicels 141–157 × 15–20 μm, swollen at base.

##### Specimens examined.

China, • Sichuan, Liangshan Yi Autonomous Prefecture, Meigu County, on *Rosa omeiensis* var. *pteracantha* Rehder & E.H. Wilson, 6 Oct. 1989, Jianyun Zhuang (HMAS64407 (holotype), HMAS64408, HMAS64411).

##### Notes.

Morphologically similar to *P. cinnamomeum*, this species differs in having noticeably broader teliospores (32–38 μm vs. 24–29 μm in *P. cinnamomeum*). The number of teliospore cells (5–6) is comparable between the two species. Phylogenetic analysis places *P. robustum* in a distinct clade (Fig. 1), supporting its recognition as a separate species.

#### Phragmidium rosae

Taxon classificationFungiPuccinialesPhragmidiaceae

- davuricae Miura, 1928, Flora of Manchuria and East Mongolia 3: 374.

159C811A-30CF-5186-B883-018B38444C1B

##### Description.

Spermogonia and aecia not seen. Uredinia hypophyllous, 0.2–0.4 mm diam.; urediniospores 17–24 × 15–19 μm, echinulate. Telia hypophyllous, black, 0.2–0.5 mm diam.; teliospores 5–6-celled, 56–81 × 23–28 μm, coarsely verrucose, apical papillae 7–19 μm long; pedicels 56–96 × 10–25 μm, swollen at base.

##### Specimens examined.

China, • Xinjiang, Ili Kazakh Autonomous Prefecture, Gongliu County, Kurdning Nursery, on *Rosa oxyacantha* M. Bieb., 29 Jul. 2011, Ying Mei Liang, Chengming Tian, Rong Ma (BJFC-R00651, BJFC-R00670); • Gansu, Zhangye City, Sunan Yugur Autonomous County, Horseshoe Temple, on *Rosa* sp., 17 Aug. 2018, Bin Cao (BJFC-R03555). For a complete list of all specimens examined, see Suppl. material 1.

##### GenBank numbers.

ITS: MW729587 (BJFC-R00651), MW729588 (BJFC-R00670), MW729589 (BJFC-R03555); LSU: MW729511 (BJFC-R00651), MW729512 (BJFC-R00670), MW729513 (BJFC-R03555).

##### Notes.

This species is morphologically similar to *P. tuberculatum*, with 5–6-celled teliospores (56–81 × 23–28 μm), and has long been considered a synonym of *P. tuberculatum* by some authors (Azbukina 1984; Zhuang et al. 2012). However, the two species can be distinguished by the width of the teliospores and the surface ornamentation of the urediniospores. The teliospores of *P. tuberculatum* are 27–35 μm wide (Deutsche 1883), whereas those of *Phragmidium rosae*-*davuricae* are narrower, reaching a maximum width of only 28 μm. Furthermore, *P. tuberculatum* possesses urediniospores with conspicuous and somewhat larger spines (Deutsche 1883; Liu 2021). Phylogenetic analysis reveals that they occupy separate, well-supported clades (Fig. 1), confirming their status as distinct species (Liu 2021).

#### Phragmidium rosae

Taxon classificationFungiPuccinialesPhragmidiaceae

- multiflorae Dietel, Hedwigia 44: 132. 1905

C9D865E0-6B09-53C0-970F-BD7A6832D83E

##### Description.

Spermogonia and aecia not seen. Uredinia hypophyllous, 0.1–0.3 mm diam.; urediniospores 18–22 × 16–18 μm, densely echinulate. Telia hypophyllous, black, 0.3–0.6 mm diam.; teliospores 5–7-celled, 62–81 × 23–25 μm, coarsely verrucose, apical papillae absent or up to 5 μm long; pedicels 73–93 × 13–17 μm, swollen from middle to lower part.

##### Specimens examined.

China, • Jiangxi, Ganzhou City, Zhanggong District, Fengshan National Forest Park, on *Rosa multiflora* Thunb. var. cathayensis Rehd., 23 Jul. 2018, Yingmei Liang and Yun Liu (BJFCR03542, BJFCR03544). For a complete list of all specimens examined, see Suppl. material 1.

##### GenBank numbers.

ITS: MW729591 (BJFCR03542), MN264721 (BJFCR03544); LSU: MW729515 (BJFCR03542), MN264739 (BJFCR03544).

##### Notes.

First described from Japan on *Rosa multiflora* (Dietel 1905), this species is characterized by its distinctive pedicels: subequal in length to the teliospores, pale brown above, and gradually becoming hyaline downward, with the swollen portion occupying approximately half of the pedicel length (Liu 2021). Phylogenetic analysis shows that Chinese specimens form a well-supported clade with sequences from Japan (Fig. 1), indicating a genetically cohesive population across East Asia.

#### Phragmidium tuberculatum

Taxon classificationFungiPuccinialesPhragmidiaceae

Jul. Müll., Ber. dt. bot. Ges. 3: 391. 1885

560BB2FA-ED24-5CB7-83EE-F0A493CB2C2F

##### Description.

Spermogonia not observed. Aecia, uredinia, and telia mixed. Aecia hypophyllous, orange-yellow; aeciospores 25–30 μm diam., echinulate. Uredinia hypophyllous; urediniospore 19–22 × 15–19 μm, echinulate. Telia hypophyllous, black; teliospores 5–7-celled, 60–100 × 23–36 μm, coarsely verrucose, apical papillae 5–18 μm long; pedicels 52–136 × 14–26 μm, swollen at base.

##### Specimens examined.

China, • Qinghai, Golog Tibetan Autonomous Prefecture, Gade County, Gade Nursery, on *Rosa rugosa* Thunb., 8 Jul. 2019, Yingmei Liang, Chengming Tian, Yun Liu, Cheng Peng (BJFC-R03682-R03684); • Qinghai, Haidong City, Haidong City, Beishan Forest Farm, 22 Jul. 2013, Chengming Tian and Yang Ting (BJFC-R00951, BJFC-R00959); Inner Mongolia, • Chifeng City, Ulanbutong, on *Rosa* sp., 15 Aug. 2019, Yingmei Liang, Chengming Tian, Yun Liu (BJFC-R03333); New Zealand, • Ackland, 9 Apr. 2019, Yingmei Liang (BJFC-R03561, BJFC-R03563).

##### GenBank numbers.

ITS: MW729606 (BJFC-R00951), MW729607 (BJFC-R00959), MW729608 (BJFC-R03561), MW729609 (BJFC-R03563), MW729610 (BJFC-R03682), MW729611 (BJFC-R03683), MW729613 (BJFC-R03684), MW729613 (BJFC-R03333); LSU: MH285382 (BJFC-R00951), KP407636 (BJFC-R00959), MW729530 (BJFC-R03561), MW729531 (BJFC-R03563), MW729532 (BJFC-R03682), MW729533 (BJFC-R03683), MW729534 (BJFC-R03684), MW729535 (BJFC-R03333).

##### Notes.

This species is distinguished by two unique spore ornamentation types: urediniospores with echinulae arising from a mushroom-shaped base (gyroscopic-echinulate type) and aeciospores with echinulae arising from a plaque-like base (plaque-echinulate type) (Liu 2021). These features are not found in other *Phragmidium* species on *Rosa*. Phylogenetically, it forms a well-supported clade sister to *P. altaicum* (Fig. 1).

#### Phragmidium warburgianum

Taxon classificationFungiPuccinialesPhragmidiaceae

(Henn.) Y.M. Liang & Y. Liu Mycologia 112(4): 747. 2020

384B0552-CA30-5079-884B-EA2A2365121C

##### Description.

Spermogonia and aecia systemically infecting the host, forming witches’ brooms. Spermogonia type 2, 139–206 × 58–104 μm. Aecia aparaphysate, orange-red; aeciospores 20–29 × 12–18 μm, verrucose. Uredinia hypophyllous, orange-red; urediniospores 18–25 × 11–16 μm, echinulate. Telia hypophyllous, white; teliospores 2–4-celled, cells 21–34 × 10–17 μm, smooth.

##### Specimens examined.

China, • Zhejiang, Ningbo City, on *Rosa* sp. (B (type specimen). Japan, Ishigaki Island, on *Rosa bracteata* J.C. Wendl., 10 Dec. 1995, Y. Ono (BJFC-R03458 (= IBAR07744)), 17 Feb. 2009 (BJFC-R03459 (= IBAR10130)).

##### GenBank numbers.

ITS: MN264726 (BJFC-R03458 (IBAR07744)), MN264727 (BJFC-R03459 (IBAR10130)); LSU: MN264744(BJFC-R03458 (IBAR07744)), MN264745 (BJFC-R03459 (IBAR10130)).

##### Notes.

Originally described as *Caeoma warburgiana* based on specimens from China and later transferred to *Kuehneola* (Ono 2012), this species causes systemic infections resulting in witches’ brooms. Molecular phylogenetic analysis clearly places it within *Phragmidium* (Liu et al. 2020; Fig. 1), necessitating the new combination *P. warburgianum*. It shares type 2 spermogonia (Cummins and Hiratsuka 2003) with *P. leucoaecium* but differs in having aeciospores with regular circular verrucae (vs. irregularly elongated verrucae in *P. leucoaecium*).

#### Phragmidium zangdongii

Taxon classificationFungiPuccinialesPhragmidiaceae

Y.M. Liang & Yun. Liu, Mycological Progress 17(8): 967–988. 2018

AD5ED056-09A6-5600-8ABF-5D332E5EBB73

##### Description.

Spermogonia and uredinia not seen. Aecia hypophyllous, up to 12 mm long; aeciospores 20–23 × 17–19 μm, echinulate. Telia hypophyllous, black, 0.2–0.4 mm diam.; teliospores 11–13-celled, 82–110 × 23–31 μm, coarsely verrucose, apical papillae up to 10 μm long; pedicels 132–152 × 14–16 μm, swollen at base.

##### Specimens examined.

China, • Xizang, Tibetan Plateau Ecological Observation Station of the Chinese Academy of Sciences, on *Rosa tibetica* Yu et Ku, 24 Jul. 2016, Yingmei Liang and Jing Cao (BJFC-R02447, BJFC-R03013).

##### GenBank numbers.

ITS: MH128372 (BJFC-R02447), MH128373 (BJFC-R03013); LSU: MG669108 (BJFC-R02447), MG669109 (BJFC-R03013).

##### Notes.

Described from Xizang, China, on *Rosa tibetica* (Liu et al. 2018), this species is notable for its 11–13-celled teliospores (82–110 × 23–31 μm) and the development of uredinia on fruits—an unusual trait in the genus (Liu 2021). It resembles *P. fusiforme* and *P. rosae*-lichiangensis in teliospore cell number but differs in having cylindrical teliospores (vs. fusiform in *P. fusiforme*) and longer pedicels (132–152 μm vs. 65–111 μm in *P. rosae*-lichiangensis). Phylogenetic analysis places it in a well-supported clade (Fig. 1).

#### Phragmidium zhouquense

Taxon classificationFungiPuccinialesPhragmidiaceae

Y.M. Liang & T. Yang, Phytotaxa 217(2): 185. 2015

094473B3-6221-57E5-A264-687842739D72

##### Description.

Spermogonia and uredinia not seen. Aecia amphigenous, 0.5–1.5 mm diam.; aeciospores 13–20 × 13–16 μm, annulate. Telia hypophyllous, brown, 0.5–2.5 mm diam.; teliospores 6–8-celled, 67–103 × 32–39 μm, coarsely verrucose, apical papillae 3.5–6 μm long; pedicels 80–160 × 14–24 μm, swollen at base.

##### Specimens examined.

China, • Yunnan, Diqing Tibetan Autonomous Prefecture, Shangri-La City, Nixi Village, on *Rosa omeiensis* Rolfe., 23 Jul. 2016, Yingmei Liang, Jing Cao (BJFC-R02390, BJFC-R02395); • Gansu, Gannan Tibetan Autonomous Prefecture, Zhouqu County, Zhouqu Hanban Forest Farm, 20 Aug. 2014, Yingmei Liang, Bin Cao (BJFC-R01516, BJFC-R01529); • Xizang, Nyingchi City, Bayi District, Kadingou Scenic Area, on *R. mairei* Levl.,23 Jul. 2016, Yingmei Liang and Bin Cao (BJFCR02412).

##### GenBank numbers.

ITS: MN264728 (BJFC-R01516), MN264729 (BJFC-R01529), MW729620 (BJFC-R02390), MW729621 (BJFC-R02395), MW729622 (BJFC-R02412); LSU: MN264746 (BJFC-R01516), MN264747 (BJFC-R01529), MW729542 (BJFC-R02390), MW729543 (BJFC-R02395), MW729544 (BJFC-R02412).

##### Notes.

First described from Gansu, China, on *Rosa omeiensis* (Yang et al. 2015), this species is characterized by 6–8-celled teliospores (67–103 × 32–39 μm) and aeciospores with annulate surface ornamentation (Liu 2021). Although its teliospores resemble those of *P. mucronatum*, the urediniospores lack a pore membrane and bear annulate ornamentation (vs. having a pore membrane and being echinulate in *P. mucronatum*). Phylogenetic analysis reveals a well-supported sister relationship with *P. cinnamomeum* (Fig. 1).

### Key to species of *Phragmidium* occurring on *Rosa*

This key is primarily based on morphological characters observed in the available specimens. Because several *Phragmidium* species on *Rosa* exhibit overlapping teliospore dimensions and incompletely known life cycles, species identification should, where necessary, be confirmed using host association and molecular phylogenetic evidence.

**Table:** 

1	Teliospores not observed in available material	**2**
–	Teliospores present	**4**
2	Uredinia absent; spermogonia and aecia present	**3**
–	Uredinia present; telia absent	*** P. jiangxiense***
3	Aecia white to yellowish; aeciospores with irregularly elongated verrucae; spermogonia type 2	*** P. leucoaecium***
–	Aecia orange-red; infection systemic, producing witches’ brooms	*** P. warburgianum***
4	Teliospore wall smooth	**5**
–	Teliospore wall verrucose or sparsely verrucose	**6**
5	Teliospores 2–5-celled; pedicels shorter than 10 μm	*** P. japonicum***
–	Teliospores 2–4-celled; often producing witches’ brooms	*** P. warburgianum***
6	Teliospores consistently 2-celled; pedicels atrophied or degenerated	*** P. kamtschatkae***
–	Teliospores with more than two cells; pedicels developed	**7**
7	Telia developing on fruits and branches, forming large crust-like sori; teliospores 12–16-celled	*** P. handelii***
–	Telia mainly on leaves; teliospores not as above	**8**
8	Teliospores usually with 9 or more cells	**9**
–	Teliospores usually with fewer than nine cells, or 7–10-celled but shorter than 75 μm	**12**
9	Teliospores fusiform, tapering toward both ends	*** P. fusiforme***
–	Teliospores cylindrical	**10**
10	Teliospores 11–13-celled; pedicels 132–152 μm long; uredinia occasionally developing on fruits	*** P. zangdongii***
–	Teliospores 9–11-celled; pedicels shorter than 120 μm	**11**
11	Teliospores 85–122 × 21–30 μm; urediniospores densely echinulate	*** P. yangiae***
12	Urediniospores with conspicuous germ-pore caps	*** P. mucronatum***
–	Urediniospores without conspicuous germ-pore caps	**13**
13	Aeciospores with annulate ornamentation	*** P. zhouquense***
–	Aeciospores absent, unknown, or not annulate	**14**
14	Urediniospore echinulae arising from plaque-like bases	*** P. shennongjiaense***
–	Urediniospore echinulae not arising from plaque-like bases	**15**
15	Aeciospores candle-echinulate; urediniospore echinulae arising from mushroom-shaped bases	*** P. altaicum***
–	Aeciospores not candle-echinulate; urediniospores otherwise ornamented	**16**
16	Urediniospore echinulae arising from mushroom-shaped basal structures, gyroscopic-echinulate type	*** P. tuberculatum***
–	Urediniospores densely or simply echinulate	**17**
17	Teliospores 47–72 × 19–24 μm, 7–10-celled; pedicels weakly swollen in the lower half	*** P. xinjiangense***
–	Teliospores usually longer than 60 μm; other characters not as above	**18**
18	Teliospores subfusiform, widest near the middle	*** P. montivagum***
–	Teliospores cylindrical	**19**
19	Telia and teliospores chestnut-brown; teliospores mostly 5–6-celled	*** P. cinnamomeum***
–	Telia black or brown at maturity; teliospores not chestnut-brown	**20**
20	Teliospores broad, 32–38 μm wide; apical papillae 1–4 μm long	*** P. robustum***
–	Teliospores usually narrower than 32 μm	**21**
21	Teliospores 70–105 × 25–35 μm; apical papillae up to 12 μm long	*** P. butleri***
–	Teliospores usually narrower or shorter; characters not as above	**22**
22	Apical papillae absent or up to 5 μm long	**23**
–	Apical papillae usually longer than 5 μm	**24**
23	Teliospores 67–87 × 22–30 μm, 7–9-celled	*** P. chayuensis***
–	Teliospores 62–81 × 23–25 μm, 5–7-celled; pedicels swollen from the middle to lower part	*** P. rosae-multiflorae***
24	Teliospores 56–81 × 23–28 μm, predominantly 5–6-celled; apical papillae 7–19 μm long	*** P. rosae-davuricae***

## Discussion

### Diversity of aecial stages

Compared with telial morphology, aecial stages have been less frequently used in the taxonomy of *Phragmidium* (Müller 1886; Sydow and Sydow 1915; Wei 1988; Wahyuno 2002). In the present study, aecia were not available for several taxa, including the two new species. Nevertheless, the specimens with aecial stages show that aeciospore characters may be useful for distinguishing some species. Differences were found mainly in aeciospore shape and wall ornamentation, particularly in the structure of the spore wall. These observations are consistent with earlier treatments in which aeciospore morphology was considered informative for rust taxonomy (Müller 1886; Sydow and Sydow 1915; Wei 1988; Wahyuno 2002). However, the present material also shows the limitations of using aecial characters alone. Some characters could not be examined in all taxa, and the available collections were unevenly distributed among species. Therefore, aecial morphology should be used together with telial characters and molecular evidence rather than treated as an independent basis for species delimitation. The number and distribution of germ pores may have taxonomic value, but these characters were not consistently observed in the available specimens. Fresh collections, together with scanning electron microscopy when possible, will be needed to determine whether these characters are stable within species and whether they correspond to phylogenetic lineages (Vilgalys and Hester 1990; Ono 2019).

### Diversity of telial stages

The telial stage remains the main source of morphological characters for species recognition in *Phragmidium* (Müller 1886; Sydow and Sydow 1915; Cummins and Hiratsuka 2003; Zhuang et al. 2012). The species examined here differ in teliospore shape, number of cells, wall ornamentation, apical papillae, and pedicel morphology. Teliospore shape and septation have long been used in species identification, but these characters overlap among several taxa. They are therefore useful for preliminary identification but are not always sufficient for delimiting species. Wall ornamentation, apical papillae, and pedicel morphology provided additional characters for separating closely similar taxa. In several cases, these characters were also consistent with the lineages recovered in the combined ITS+LSU phylogeny (Müller 1886; Wei 1988; Sun et al. 2022). This supports the continued use of telial morphology in *Phragmidium* taxonomy but also shows that no single character is reliable across all species. Similar difficulties have been noted in recent studies of rust fungi (Liu et al. 2018; Sun et al. 2022). Pedicel hygroscopicity was not examined in this study because suitable fresh material was unavailable. This character has not been fully evaluated in Chinese *Phragmidium* species and deserves further study. Overall, the results support a combined approach in which telial morphology, aecial characters when available, host information, and molecular phylogenetic evidence are considered together.

### Putative host preference

Host association has been widely used in *Phragmidium* taxonomy because many species appear to occur on particular genera or species of *Rosaceae* (Wei 1988; Cummins and Hiratsuka 2003; Zhuang et al. 2012; Liu et al. 2018; Liu 2021; Sun et al. 2022). All specimens examined in this study were collected from *Rosa* species, including *R. albertii*, *R. rugosa*, *R. multiflora*, *R. omeiensis*, and *R. laevigata*. The results suggest that host information is helpful for identification, especially when combined with morphology, but it should not be used alone to define species. The relationship between host association and species boundaries was not always simple. Morphologically similar taxa may occur on different *Rosa* hosts, whereas distinct phylogenetic lineages may occur on the same or closely related hosts. For example, *Phragmidium shennongjiaense* and *P. xinjiangense* both occur on *Rosa*, but they differ in teliospore morphology and were recovered as separate lineages in the combined ITS+LSU phylogeny. Similar discordance between host association and phylogenetic placement has also been reported in other rust fungi (Liu et al. 2018, 2019, 2020, 2021; Liu 2021; Zhao et al. 2021; Sun et al. 2022).

The host records reported here should therefore be regarded as putative host preferences rather than confirmed host specificity. This is because the study was based on field collections and herbarium specimens, and no cross-inoculation experiments were conducted. Whether the species treated here are strictly restricted to particular *Rosa* hosts or have broader host ranges remains unresolved. More intensive sampling across host species, together with cross-inoculation or pathogenicity experiments, will be required to test host specificity in *Phragmidium*.

### Geographic distribution

The specimens examined in this study show that *Rosa*-associated *Phragmidium* species occur across a wide geographic range in China, including Xinjiang, Xizang, Qinghai, Gansu, Hubei, Yunnan, Fujian, Jiangxi, and Beijing. This broad distribution is consistent with previous views that East Asia is an important region for the diversity of *Phragmidium* (Hiratsuka et al. 1992; Wei 1988; Cao and Li 1999; Yun et al. 2011; Zhuang et al. 2012; Liu 2021; Liu et al. 2021; Zhang et al. 2025). Some species appear to be geographically restricted in the available material. *Phragmidium xinjiangense* is currently known from Xinjiang and Xizang, whereas *P. shennongjiaense* has been collected from Hubei, Qinghai, and Gansu. By contrast, *P. rosae-multiflorae* and *P. tuberculatum* have been recorded from broader areas (Wei 1988; Liu et al. 2018; Liu 2021; Sun et al. 2022). These differences may indicate real variation in distributional ranges, but they may also reflect uneven collecting intensity. Many regions of China, especially mountainous areas with abundant wild *Rosa* species, remain insufficiently sampled.

The recognition of two new species in the present study shows that the diversity of *Phragmidium* in China is still not fully known. This agrees with recent studies showing that additional rust fungal diversity continues to be discovered in East Asia (Wei 1988; Liu et al. 2018; Zhao et al. 2021; Sun et al. 2022; Zhang et al. 2025). Western and southwestern China are likely to be especially important because of their complex topography, diverse habitats, and rich *Rosa* flora. Further collections from these regions, together with multilocus phylogenetic analyses, will help clarify the diversity, distribution, and evolutionary history of *Phragmidium* in China.

## Supplementary Material

XML Treatment for Phragmidium shennongjiaense

XML Treatment for Phragmidium xinjiangense

XML Treatment for Phragmidium yangiae

XML Treatment for Phragmidium altaicum

XML Treatment for Phragmidium butleri

XML Treatment for Phragmidium chayuensis

XML Treatment for Phragmidium cinnamomeum

XML Treatment for Phragmidium fusiforme

XML Treatment for Phragmidium handelii

XML Treatment for Phragmidium japonicum

XML Treatment for Phragmidium jiangxiense

XML Treatment for Phragmidium kamtschatkae

XML Treatment for Phragmidium leucoaecium

XML Treatment for Phragmidium montivagum

XML Treatment for Phragmidium mucronatum

XML Treatment for Phragmidium robustum

XML Treatment for Phragmidium rosae

XML Treatment for Phragmidium rosae

XML Treatment for Phragmidium tuberculatum

XML Treatment for Phragmidium warburgianum

XML Treatment for Phragmidium zangdongii

XML Treatment for Phragmidium zhouquense

## Additional information

### Artificial Intelligence (AI) use

The authors accept full responsibility for the content of the manuscript, including the disclosure of any use of AI.

No AI tools were used in the preparation of this manuscript.

### Author ORCIDs

Y. Liu https://orcid.org/0000-0002-1473-2291

N. Jiang https://orcid.org/0000-0002-9656-8500

Y. Ono https://orcid.org/0000-0002-8247-7245

Y. Liang https://orcid.org/0000-0002-1690-5512

### Data availability

All of the data that support the findings of this study are available in the main text or Supplementary Information.

## Supplementary materials

Supplementary material 1 Specimens of *Phragmidium* species on *Rosa* examined in this study, with collection information and GenBank accession numbers for sequenced specimens https://doi.org/10.3897/mycokeys.138.201984.suppl1 Data type xlsx https://binary.pensoft.net/file/1740233This dataset is made available under the Open Database License (http://opendatacommons.org/licenses/odbl/1.0/). The Open Database License (ODbL) is a license agreement intended to allow users to freely share, modify, and use this Dataset while maintaining this same freedom for others, provided that the original source and author(s) are credited. Yun Liu, Zelin Zhao, Zhimin Du, Xiuhan Xing, Ning Jiang, Yoshitaka Ono, Yingmei Liang
